# Determination of Gas Permeation Properties in Polymer Using Capacitive Electrode Sensors

**DOI:** 10.3390/s22031141

**Published:** 2022-02-02

**Authors:** Jaekap Jung, Gyunghyun Kim, Gahyoun Gim, Changyoung Park, Jihun Lee

**Affiliations:** 1 Hydrogen Energy Materials Research Center, Korea Research Institute of Standards and Science, Daejeon 34113, Korea; ljh93@kriss.re.kr; 2 Department of Physics and Research Institute of Natural Science, Gyeongsang National University, Jinju 52828, Korea; 2016010673@gnu.ac.kr (G.K.); cutegosum@gnu.ac.kr (G.G.); parkcy@gnu.ac.kr (C.P.)

**Keywords:** polymer, gas permeation, diffusion, capacitance, kinetic diameter

## Abstract

The objective of this work was to develop an effective technique for characterizing the permeation properties of various gases, including H_2_, He, N_2_, and Ar, that are absorbed in polymers. Simultaneous three-channel real-time techniques for measuring the sorption content and diffusivity of gases emitted from polymers are developed after exposure to high pressure and the subsequent decompression of the corresponding gas. These techniques are based on the volumetric measurement of released gas combined with the capacitance measurement of the water content by both semi-cylindrical and coaxial-cylindrical electrodes. This minimizes the uncertainty due to the varying temperature and pressure of laboratory environments. The gas uptake and diffusivity are determined as a function of the exposed pressure and gas spices in nitrile butadiene rubber (NBR) and ethylene propylene diene monomer (EPDM) polymers. The pressure-dependent gas transport behaviors of four different gases are presented and compared with those obtained by different techniques. A linear correlation between the logarithmic diffusivity and kinetic diameter of molecules in the gas is found between the two polymers.

## 1. Introduction

The permeability of a polymer is defined as the rate at which it is penetrated by various gases. The characteristic passage of gas through a polymer is affected by the solubility in the polymer, and gases pass through the polymer sheet by the process of diffusion. In other words, gas permeation is the passage of a permeant through a polymer material. The process of permeation involves the diffusion of molecules—i.e., the permeant—through a membrane or interface where the permeant will move from a high concentration to a low concentration across the interface. Permeation is extensively utilized for various applications, such as in the food packaging field, tires and fuel cells in automobiles, electrical insulating materials, the medical field for drug delivery, thermoplastic piping in gas transportation, and O-rings in high-pressure gas vessels [1,2,3,4,5]. Studying the permeability characterization of materials with different gases and under different environmental conditions is crucial in order to understand whether the corresponding material is adapted to the chosen gases. At the same time, the transport properties of gases to permeate the materials can be clarified with reliable measurement techniques.

Meanwhile, the gas permeation of a material can be measured by numerous methods that quantify the permeability of a material. These methods include manometric methods [6,7,8], constant-pressure methods [7], gravimetric techniques [9], magnetic suspension balance methods [10,11], gas chromatography [12], and computer simulation [13,14]. Most methods are time-consuming, requiring complicated processes and fine control. For instance, for polymers with a diffusivity in the order of 10^−11^ m^2^/s and with a thickness above 3 mm, it takes at least a few days to reach the adsorption/desorption equilibrium and then complete the permeation measurement. Furthermore, the variation in both temperature and pressure across the days affects measurements of aspects such as the gas volume and then increases the uncertainty in the determination of permeation parameters. Thus, the instability due to temperature and pressure should be minimized to achieve precise measurement and compensation.

Effective and real-time automatic measurements are required to overcome the limitations of methods and further enhance the reliability of the measurement of permeability characteristics. We sought to find an appropriate technique for determining the permeation properties of several gases dissolved in materials. Thus, we developed the volumetric analysis technique (VAT) in previous studies [15] and confirmed this by comparing the results obtained using VAT with those obtained using different methods, such as gas chromatography (GC) by thermal desorption analysis (TDA) and gravimetric measurement by electronic balance for same samples. The results were found to be consistent with each other. A more effective technique is to combine a volumetric measurement using a graduated cylinder and automatic capacitance measurement with electrodes through a frequency response analyzer interfaced with a PC. The developed technique reduces the uncertainty of permeation parameters due to the varying temperature and pressure of the laboratory environment. The techniques were applied to nitrile butadiene rubber (NBR) and ethylene propylene diene monomer (EPDM) polymers, which are used for gas sealing materials under high pressure. The solubility, diffusivity, and permeability of the four different gases in the two polymers were investigated as a function of the exposed pressure and compared with those determined by different methods. The permeation characteristics obtained by this method were described. Another motivation of our research was that the polymer materials can be applicable for various gas sealing requirements under a high pressure. The diffusivity in the NBR and EPDM polymers can be interpreted in terms of the kinetic diameter of molecules in the employed gases.

## 2. Experimental Aspects

### Sample Preparation and Gas Exposure Conditions

The compositions and densities of the NBR and EPDM polymer specimens used in this study have already been listed in previous literature [15,16]. NBR samples with two different thicknesses and EPDM samples with different shapes/dimensions were used: cylindrical-shaped NBR samples with a radius of 7.0 mm and thicknesses of 1.1 mm and 2.2 mm were prepared. Cylindrical-shaped EPDM samples with a radius of 7.0 mm and thicknesses of 1.4 mm and 2.5 mm as well as spherical-shaped EPDM with a radius of 4.9 mm were also prepared.

A SUS 316 chamber with an inner diameter of 50 mm and height of 90 mm was used for gas exposure to high pressure at room temperature and a specified pressure. The chamber was purged three times with the corresponding gas of 1 MPa–3 MPa depending on the pressure before the gas exposure. We exposed the specimen to the gas for 24 h in a pressure range from 1.5 MPa to 10 MPa. Gas charging for 24 h is sufficient to attain the equilibrium state for gas sorption, except for N_2_ gas exposure. N_2_ gas charging for 48 h is needed to attain the equilibrium state for N_2_ sorption because of its slow diffusion rate. After exposure to gas, the valve was opened and the gas in the chamber was released. After decompression, the elapsed time was recorded from the moment (*t* = 0) at which the high-pressure gas in the chamber was reduced to atmospheric pressure when the time was set to zero. Since the specimen was loaded in the graduated cylinder after decompression, it took approximately 5–10 min to start the measurement. The gas content emitted for the inevitable time lag could be measured later by offset determination via the simulation.

## 3. Two Types of Capacitor Electrodes to Measure the Water Level

We employed two types of electrodes to measure the capacitance corresponding to the water content in the acrylic tube (graduated cylinder). A semi-cylindrical capacitor and coaxial-cylindrical capacitor electrodes were fabricated and attached to the outer wall of the graduated cylinder. The capacitance was measured at 1 MHz with two electrodes by a frequency response analyzer (VSP 300) with a general-purpose interface bus (GPIB) connected to a PC.

### 3.1. Semi-Cylindrical Capacitor Electrode

**Figure 1:**
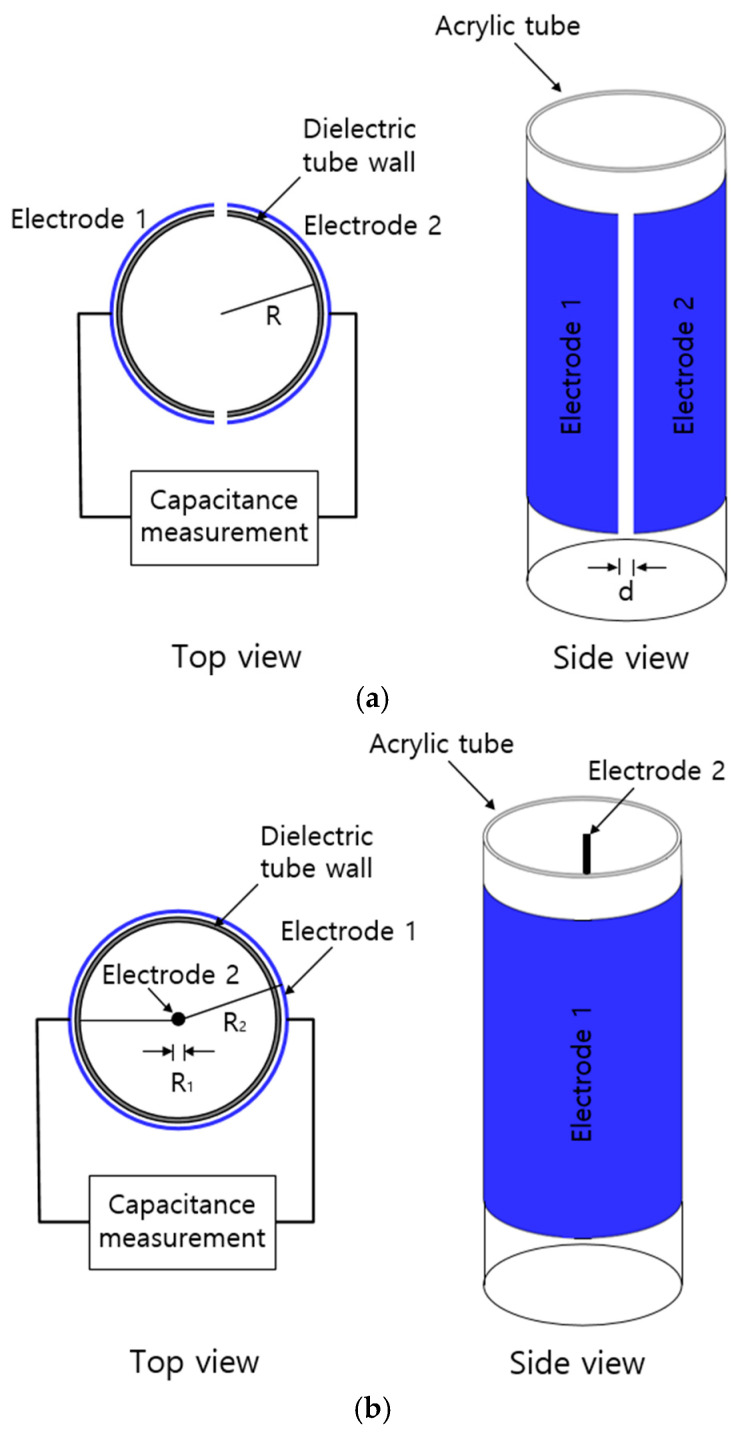
(**a**) Configuration of the semi-cylindrical capacitor electrode, indicated in blue. (**b**) Configuration of the coaxial-cylindrical capacitor electrode.

The capacitive sensor fabricated with semi-cylindrical electrodes mounted outside of an acrylic tube is shown in Figure 1a. An acrylic tube surrounded by two semi-cylindrical electrodes is filled with water gas. The electrode attached to the outer wall of the acrylic tube is made of copper cylinder with a thickness of 1 mm. The capacitance of the sensor depends on the dielectric permittivity of the medium existing between the electrodes. The dielectric permittivity of water is 78.4 times larger than that of gas inside the graduated cylinder. Thus, the position shift of the water level in the two electrodes leads to a change in the capacitance.

The actual capacitance (*C_a_*) due to water gas is connected in series with the capacitance (*C_tw_*) of the acrylic dielectric tube wall. The total capacitance (*C_t_*) between the semi-cylindrical electrodes can be expressed as:

(1) $$C_{t}=\frac{C_{a}C_{tw}}{C_{a}+C_{tw}}$$

The actual permittivity ($\varepsilon_{a})$ of both the water and gas inside the cylinder, depending on the volume fraction of the two media, is given by:

(2) $$\varepsilon_{a}=\frac{V_{w}\varepsilon_{w}+V_{0}\varepsilon_{0}}{V_{t}}$$

where $V_{w}$ is the water volume in the cylinder, $\varepsilon_{w}$ is the dielectric permittivity of water, $V_{0}$ is the gas volume in the cylinder, $\varepsilon_{0}$ is the dielectric permittivity of gas, and $V_{t}$ is the total volume.

The actual capacitance with two semi-cylindrical electrodes of the same size is calculated as [17]:

(3) $$C_{a}=\sum_{i=0}^{n}2\varepsilon^{*}{}_{0}\varepsilon_{a}A\times \left[\frac{1}{d+\left(i-1\right)\Delta d}\right]+\frac{\varepsilon_{0}\varepsilon_{a}A}{2R}$$

where *A* is the area of the electrode, $\varepsilon^{*}{}_{0}$ is the dielectric permittivity of free space, *d* is the distance between the electrodes, *R* is the radius of the acrylic tube, and Δd is an increment distance between semi-cylindrical concave electrodes. In this work, the values in Equation (3) are constant except for $\varepsilon_{a}$. The capacitance values with respect to the water content are obtained by a combination of Equations (1)–(3). We measured the change in actual capacitance ($C_{a})$ $\mathrm{by}\;\mathrm{the}\;\mathrm{change}\;\mathrm{in}\;\varepsilon_{a}$ arising from the changing water level in the graduated cylinder. Therefore, the changing water level corresponding to the change capacitance is determined with the precalibration equation between the capacitance and water level, which will be presented in the following chapter.

### 3.2. Coaxial-Cylindrical Capacitor Electrode

Another capacitive sensor is designed with coaxial-cylindrical electrodes mounted at the center and outside of an acrylic tube, as shown in Figure 1b. The water gas in the acrylic tube is filled between two coaxial electrodes. The change in capacitance, ΔC, with respect to the water level, *h*, and remaining height, *L−h*, in the cylinder filled with gas is given by [18]:

(4) $$\Delta C=\frac{2\pi \varepsilon_{0}\left(\varepsilon_{w}h+\varepsilon_{g}\left(L-h\right)\right)}{\ln\left(\frac{R_{2}}{R_{1}}\right)}=\frac{2\pi \varepsilon_{0}\left(\varepsilon_{w}-\varepsilon_{g}\right)h}{\ln\left(\frac{R_{2}}{R_{1}}\right)}+\frac{2\pi \varepsilon_{0}\varepsilon_{g}L}{\ln\left(\frac{R_{2}}{R_{1}}\right)}$$

where *h* is the water level, *L* is the length of the cylindrical capacitor, *R*_1_ is the radius of the solid cylindrical conductor (electrode 2) made of thin copper wire, and *R*_2_ is the inner radius of the coaxial cylindrical shell (electrode 1) made of copper plate. $\varepsilon_{0,\;}\varepsilon_{w},\;$and $\varepsilon_{g}$ are the permittivity of free space, water, and gas, respectively.

For a fixed configuration of the coaxial cylindrical electrode, Equation (4) indicates that ΔC is linearly related to the change in the water level, *h*. Similar to the semi-cylindrical electrode, we thus determined the water level by measuring the change in capacitance with a precalibration equation.

## 4. Volumetric Analysis Measurement System

### 4.1. Volumetric Measurement of Emitted Gas

**Figure 2:**
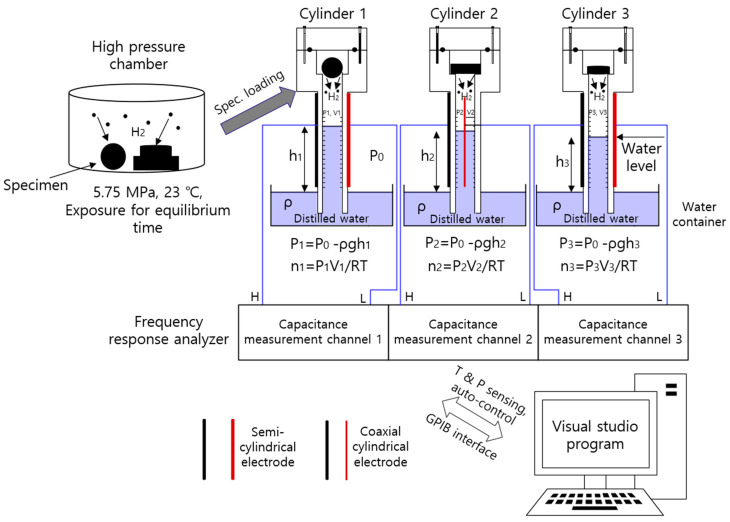
Schematic diagram of the three-channel volumetric measurement system in which three cylinders are standing. The blue part indicates the distilled water filling the water containers and cylinders. A frequency response analyzer GPIB interfaced with a PC at three channels is employed for automatic real-time capacitance measurement with both semi-cylindrical and coaxial-cylindrical electrodes.

Figure 2 shows a three-channel volumetric measurement system with three graduated cylinders and three electrodes to measure the released gas in real time. After exposure to the high-pressure chamber and subsequent decompression, the specimen is loaded into the gas space of a graduated cylinder. Three parallel standing graduated cylinders partially immersed in each water container collect and measure the gas released from the specimen. The semi-cylindrical and coaxial-cylindrical electrodes, connected in parallel to the responding capacitance measurement channel of the frequency response analyzer, are mounted outside of acrylic tubes in the left and right cylinders and center cylinder. The precise frequency response analyzer (FRA, VSP 300) with an excellent performance is a general purpose interface bus (GPIB) interfaced with a programmed PC with autosensing and autocontrol functions for the temperature and pressure. The FRA GPIB interfaced with the PC at three channels is employed for automatic real-time capacitance measurement with both semi-cylindrical and coaxial-cylindrical electrodes, as shown in Figure 2. The temperature and pressure measured near the sample are automatically applied for the calculation of the gas uptake.

The pressures ($P_{1},\;P_{2},\;\mathrm{and}\;P_{3}$) inside each graduated cylinder for the three channels are expressed as [15]:

(5) $$P_{1}=P_{o}-\rho gh_{1},\;P_{2}=P_{o}-\rho gh_{2},\;P_{3}=P_{o}-\rho gh_{3}$$

where $P_{o}$ is the outside atmospheric pressure of the cylinder, ρ is the density of distilled water in the water container, and *g* is gravity. $h_{1}$, $h_{2},$ and $h_{3}\;$are the heights of the distilled water level inside the corresponding graduated cylinder measured from the water level in the water container of channel 1, channel 2, and channel 3, respectively. $V_{1},\;V_{2},\;\mathrm{and}\;V_{3}$ are the gas volumes inside the corresponding graduated cylinder filled with gas. As shown in Figure 2, the gas inside the cylinder is governed by the ideal gas equation, *PV* = *nRT*, and R is the gas constant with 8.20544 × 10^−5^ m^3^·atm/(mol·K).

The total number of moles ($n_{1}$, $n_{2}\;\mathrm{and}\;n_{3}$) of gas inside the corresponding cylinder for the three channels is expressed at specified *P* and *T* as follows:

(6) $$n_{1}=n_{1,0}+\Delta n_{1}=\frac{(P_{o}-\rho gh_{1})V_{1}}{RT},\;n_{2}=n_{2,0}+\Delta n_{2\;}=\frac{(P_{o}-\rho gh_{2})V_{2}}{RT},\;n_{3}=n_{3,0}+\Delta n_{3}=\frac{(P_{o}-\rho gh_{3})V_{3}}{RT}$$

where $n_{1,0}$, $n_{2,0},$ and $n_{3,0}$ are the initial number of moles of air already in cylinder 1, cylinder 2, and cylinder 3, respectively, before the gas emission. The gas released from the specimen after decompression lowers the water level of the cylinder. Thus, the increased number of moles ($\Delta n_{1},\;\Delta n_{2},\;\mathrm{and}\;\Delta n_{3}$) in each cylinder from emitted gas after decompression is obtained by measuring the increase in volume ($\Delta V_{1},\;\Delta V_{2},\;\mathrm{and}\;\Delta V_{3})$ in each graduated cylinder, with the lowering of the water level as follows:

(7) $$\Delta n_{1}=\frac{(P_{o}-\rho gh_{1})\Delta V_{1}}{RT},\;\Delta n_{2}=\frac{(P_{o}-\rho gh_{2})\Delta V_{2}}{RT},\;\Delta n_{3}=\frac{(P_{o}-\rho gh_{3})\Delta V_{3}}{RT}$$

The increased number of moles in each channel is converted to the corresponding mass concentration [$C_{1}\left(t\right),\;C_{2}\left(t\right),\;\mathrm{and}\;C_{3}\left(t\right)]$ of gas emitted from the rubber sample as follows:

(8) $$\begin{array}{c} C_{1}\left(t\right)\left[\mathrm{wt}\cdot \mathrm{ppm}\right]=\Delta n_{1}\left[\mathrm{mol}\right]\times \frac{m_{gas}\;\left[\frac{g}{\mathrm{mol}}\right]}{m_{sample}\left[g\right]}\times 10^{6}\\ C_{2}\left(t\right)\left[\mathrm{wt}\cdot \mathrm{ppm}\right]=\Delta n_{2}\left[\mathrm{mol}\right]\times \frac{m_{gas}\;\left[\frac{g}{\mathrm{mol}}\right]}{m_{sample}\left[g\right]}\times 10^{6}\\ C_{3}\left(t\right)\left[\mathrm{wt}\cdot \mathrm{ppm}\right]=\Delta n_{3}\left[\mathrm{mol}\right]\times \frac{m_{gas}\;\left[\frac{g}{\mathrm{mol}}\right]}{m_{sample}\left[g\right]}\times 10^{6} \end{array}$$

where $m_{gas}$ (g/mol) is the molar mass of the gas investigated. For example, for H_2_ gas, $m_{H2\;gas}$ is 2.016 g/mol. $m_{sample}$ is the mass of the specimen. By measuring the change in the water level (ΔV), we obtained an increased number of moles and thus transformed the mass concentration of the emitted gas. Therefore, the time-dependent mass concentration by released gas can be obtained by measuring the water level change, ΔV, versus the time elapsed since decompression. The water level data were transformed from the capacitance by the precalibration data of the polynomial form between the capacitance and the position of the water level.

### 4.2. Time-Dependent Emitted Gas Concentration versus Specimen Shape

The adsorption of gas under high pressure causes the release of gas dissolved in rubber after decompression to atmospheric pressure. Assuming that the adsorption and desorption of gas are diffusion-controlled processes, the emitted gas concentration $C_{E}\left(t\right)$ in the desorption process is expressed as [19,20]:

(9) $$C_{E}\left(t\right)=C_{\infty }\left[1-\frac{6}{\pi^{2}}\overset{\infty }{\underset{n=1}{\sum }}\frac{1}{n^{2}}\exp\left(-\frac{Dn^{2}\pi^{2}t}{a^{2}}\right)\right]$$

Equation (9) is the solution to Fick’s second law of diffusion for a spherical sample with an initially constant uniform gas concentration and constant concentration at the spherical surface. $C_{\infty }$ is the saturated gas mass for an infinitely long time—i.e., the total emitted mass concentration or gas uptake in the adsorption process. *D* is the diffusion coefficient of desorption. a is the radius of the spherical rubber [19,20].

Similarly, the emitted gas content $C_{E}\left(t\right)$ for a cylindrical specimen is expressed under the boundary condition—i.e., a uniform gas concentration is initially maintained and the cylindrical surfaces are kept at a constant concentration [19,20]:

(10) $$C_{E}\left(t\right)/C_{\infty }=1-\frac{32}{\pi^{2}}\times \left[\overset{\infty }{\underset{n=0}{\sum }}\frac{\exp\left\{\frac{-{\left(2n+1\right)}^{2}\pi^{2}D_{s}t}{l^{2}}\right\}}{{\left(2n+1\right)}^{2}}\right]\times \left[\overset{\infty }{\underset{n=1}{\sum }}\frac{\exp\left\{-\frac{D_{s}\beta_{n}^{2}t}{\rho^{2}}\right\}}{\beta_{n}^{2}}\right]$$

In Equation (10), l is the thickness of the cylindrical rubber sample, ρ is the radius, and $\beta_{n}$ is the root of the zero-order Bessel function. To analyze the mass concentration data, we used a diffusion analysis program developed using Visual Studio to calculate *D* and $C_{\infty }$ in Equations (9) and (10) based on least-squares regression [15,21].

### 4.3. Diffusion Parameter Analysis through Programmed Capacitance Measurement

**Figure 3:**
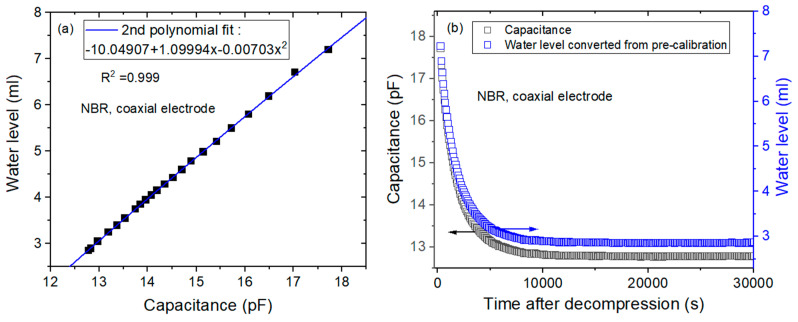

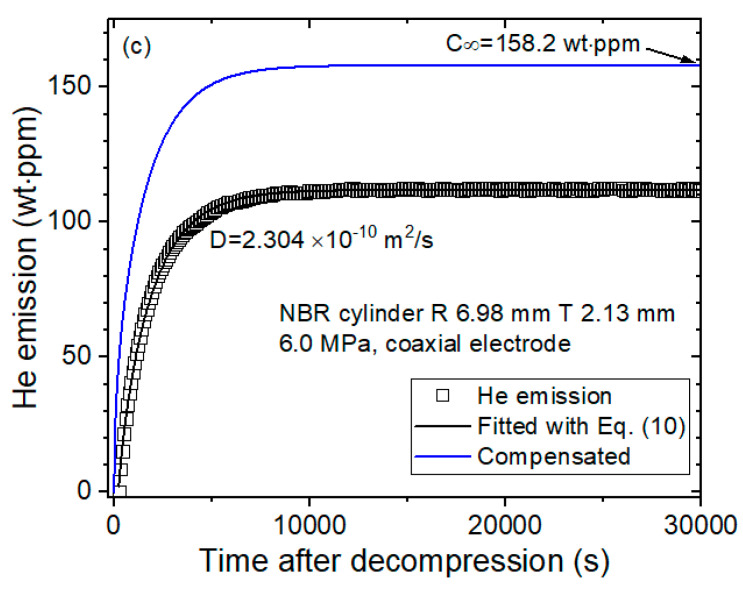
A sequence acquiring diffusion parameters measured for a NBR cylindrical rubber by employing coaxial-cylindrical electrodes in a frequency response analyzer. (**a**) Precalibration data expressed as a 2nd polynomial equation between the water level and capacitance by quadratic regression, (**b**) water level transferred from the capacitance with black and blue squares corresponding to the capacitance and transformed water level, respectively, versus time and (**c**) diffusion parameters *D* and $C_{\infty }$ determined using a diffusion analysis program by application of Equation (10). The blue line is the total compensated emission curve restoring the missing content due to the lag time.

The gas emitted from the specimen lowers the water level, and then the water level decreases as the elapsed time increases. Using programmed capacitor measurements with electrodes and diffusion analysis programs, the diffusion parameters for specimens can be determined. Figure 3a–c shows the processes used for acquiring the diffusion parameter in NBR cylindrical rubber by coaxial-cylindrical electrodes as follows:

(a) To obtain the precalibration data, the user measures the water level versus the capacitance at the corresponding channel with decreasing water levels. Then, the 2nd polynomial equation related to the position of the water level and capacitance is obtained by quadratic regression, as shown in Figure 3a. The 2nd polynomial equation originates from Equation (4). The position of the water level is measured by a digital camera.
(b) According to the precalibration data, the capacitance is transformed to the water level, as shown in Figure 3b. The black and blue squares correspond to the capacitance and position of the water level, respectively, versus the time elapsed.
(c) Last, the diffusion parameters *D* and $C_{\infty }$ are determined using a diffusion analysis program by applying Equation (10) based on least-squares regression, as shown in Figure 3c.

**Figure 4:**
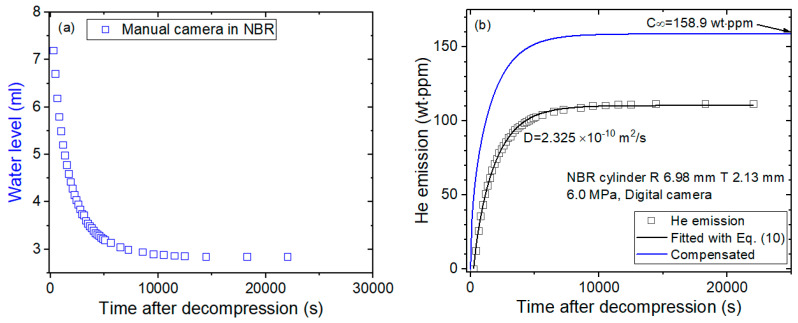
A sequence acquiring the diffusion parameter in NBR cylindrical rubber by employing a digital camera without precalibration. (**a**) Water level versus time after decompression and (**b**) diffusion parameters *D* and $C_{\infty }$ determined using a diffusion analysis program. The blue line is the total compensated emission curve restoring the missing content due to the lag time.

Figure 4 shows the sequence used for obtaining the diffusion parameter manually by a digital camera for the same NBR as Figure 3. Figure 4a shows the water level measured directly by a digital camera without precalibration, and Figure 4b shows the water level as a function of time transformed to the mass concentration, resulting in diffusion parameters *D* and $C_{\infty }$ determined using a diffusion analysis program. The two results in Figure 3 and Figure 4 are consistent with each other.

**Figure 5:**
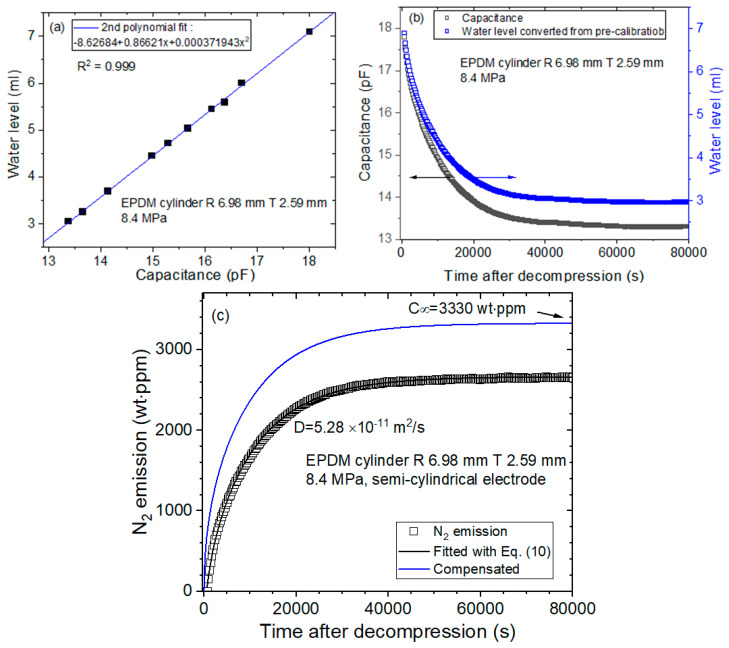
A sequence acquiring diffusion parameters measured for EPDM cylindrical rubber by employing semi-cylindrical capacitor electrodes in a frequency response analyzer. (**a**) Precalibration data expressed as a 2nd polynomial equation between the water level and capacitance by quadratic regression; (**b**) water level transformed from the capacitance, where black and blue squares correspond to the capacitance and transformed water level, respectively, versus elapsed time; and (**c**) diffusion parameters *D* and $C_{\infty }$ determined using a diffusion analysis program by the application of Equation (10). The blue line is the total compensated emission curve restoring the missing content due to the lag time.

Figure 5 represents the sequence of acquiring diffusion parameters for EPDM cylindrical rubber by employing semi-cylindrical electrodes. Figure 5a represents precalibration data expressed as the 2nd polynomial equation between the water level and capacitance by quadratic regression, which comes from Equations (1)–(3). Figure 5b shows the water level transformed from the capacitance, where the black and blue squares correspond to the capacitance and transformed water level, respectively, versus time. Figure 5c shows diffusion parameters *D* and $C_{\infty }$, which are determined using a diffusion analysis program according to Equation (10).

**Figure 6:**
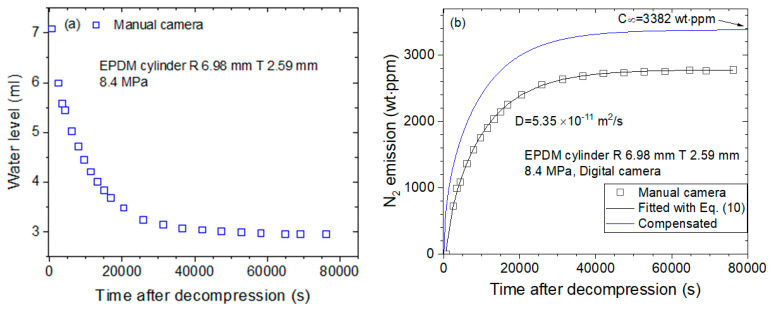
Sequence of acquiring the diffusion parameter in EPDM cylindrical rubber by employing a digital camera without precalibration. (**a**) Water level versus time after decompression and (**b**) diffusion parameters *D* and $C_{\infty }$ determined using a diffusion analysis program. The blue line is the total compensated emission curve restoring the missing content due to the lag time.

Figure 6 shows the sequence used for obtaining the diffusion parameter measured manually by a digital camera for the same EPDM as that shown in Figure 5. Figure 6a shows the water level measured directly by a digital camera, and Figure 6b shows the water level as a function of time transformed to the mass concentration, resulting in diffusion parameters *D* and $C_{\infty }$ determined using a diffusion analysis program. The two results in Figure 5 and Figure 6 are consistent with each other.

## 5. Results and Discussion

### 5.1. Stability Test of the Volumetric Measurement System

**Figure 7:**
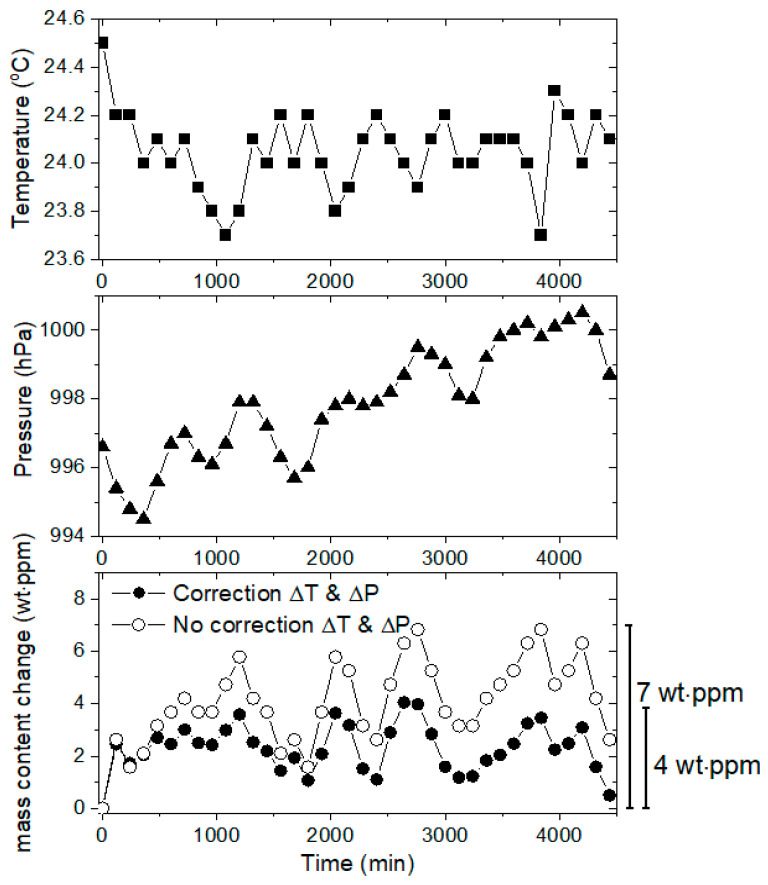
Stability for volumetric measurement with variations in the temperature and pressure over three days.

The volume and number of moles of gas in the graduated cylinder are directly affected by both the temperature and pressure in the laboratory environment. Therefore, before measuring the main diffusion properties, the stability of the volumetric measurement system should be improved by applying variations in both the temperature and pressure during long-term measurement to calculations using Equations (6) and (7). Figure 7 shows the stability measurements performed for three days, in which the temperature (top of Figure 7) and pressure (middle of Figure 7) were maintained within 24.0 ± 0.5 °C and 997.5 ± 3.5 hPa, respectively. The bottom of Figure 7 represents the stability test with (closed circle) and without (open circle) the application of variation in both the temperature and pressure to Equations (6) and (7).

The change in the mass concentration due to correction for the changes in temperature and pressure over three days is within 4 wt·ppm, which is comparable with 7 wt·ppm in the case that does not consider the variation in temperature and pressure. The system stability is improved by removing the variation in both the temperature and pressure, which are included as uncertainty factors in permeation parameter determination.

### 5.2. Pressure Dependence on the Permeation Parameter

**Figure 8:**
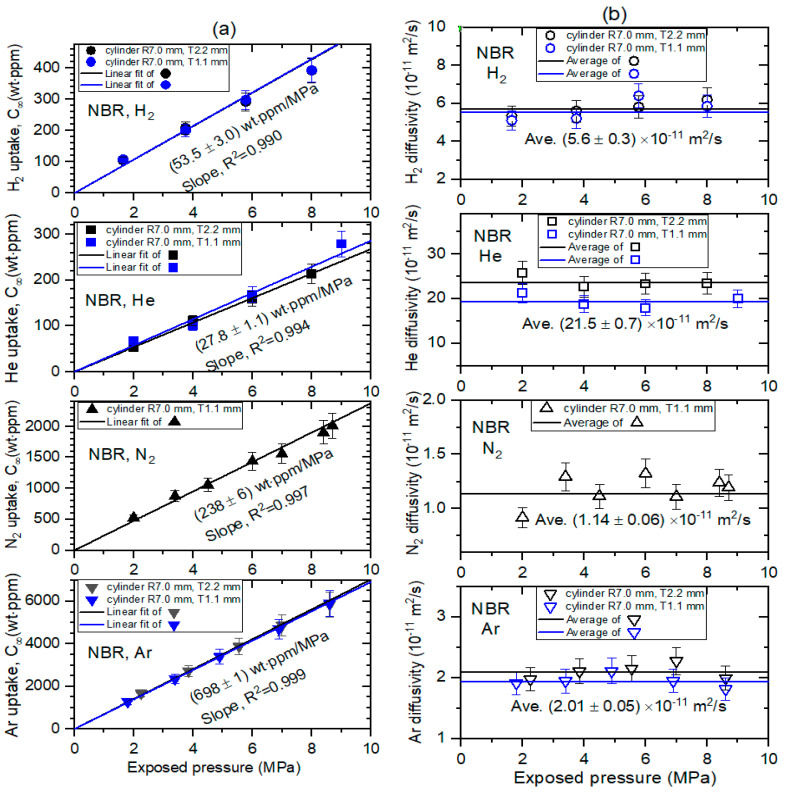
(**a**) Gas uptake ($C_{\infty }$) and (**b**) diffusivity (*D*) versus exposed pressure for four gases in cylindrical-shaped NBR with different thicknesses. R and T indicate the radius and thickness, respectively, of cylindrical-shaped NBR.

**Figure 9:**
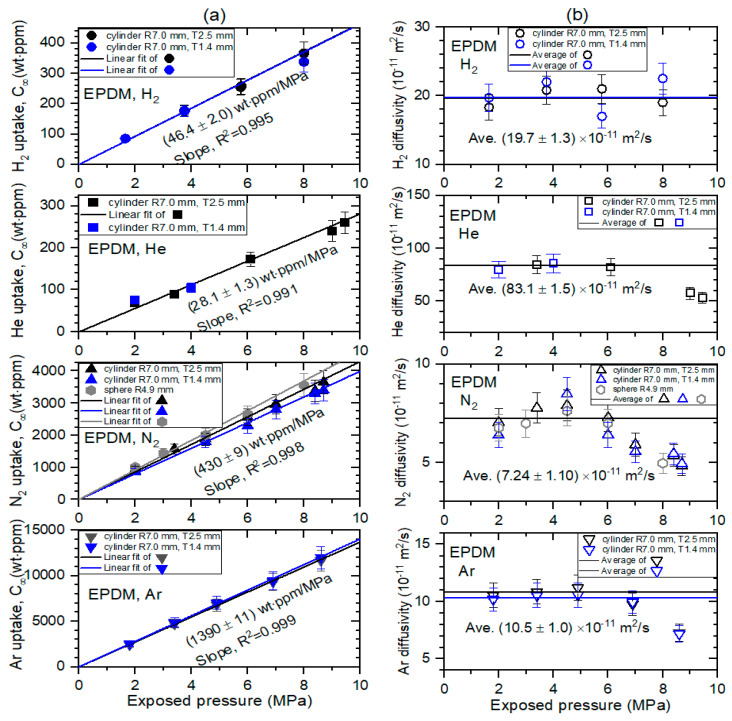
(**a**) Gas uptake ($C_{\infty }$) and (**b**) diffusivity (*D*) versus exposed pressure for four gases in cylindrical-shaped EPDM with different thicknesses and spherical-shaped EPDM. R indicates the radius of cylindrical-shaped and spherical-shaped EPDM. T indicates the thickness of the cylindrical-shaped EPDM.

Figure 8 and Figure 9 show the permeation parameters versus exposed pressure in NBR and EPDM, respectively, for four different gases with coaxial-cylindrical or semi-cylindrical electrodes at three channels. The diffusion parameters $C_{\infty }$ and *D* are determined using a diffusion analysis program by the application of Equations (9) and (10) based on least-squares regression. The standard deviation between the experimental data and the diffusion model was within 3% for both rubbers.

All the gas uptake follows Henry’s law [22] up to 9 MPa with a squared correlation coefficient R^2^ > 0.990, as indicated by the black and blue lines in Figure 8a for NBR, and black, blue, and gray lines in Figure 9a for EPDM. This implies that gas does not dissociate and penetrates into the specimen as a gas molecule. The slopes in the two specimens indicate Henry’s law of solubility. As shown in Figure 8b, the diffusivity does not represent a distinct pressure dependency. Thus, we take the average diffusivity, as indicated by the black and blue horizontal lines. Meanwhile, Figure 9b shows that the diffusivity decreases as the pressure increases above 6 MPa, except for H_2_ diffusivity. This may be ascribed to the bulk diffusion associated with the mean free path, which is normally observed for high-pressure gas diffusion. The error bars indicate the relative expanded uncertainty of 10%, as evaluated in previous research. At pressures below 6 MPa in Figure 9b, we also take the average diffusivity, as indicated by black and blue horizontal lines. As shown in Figure 8 and Figure 9, no dependence of the permeation parameters on the thickness in cylindrical-shaped NBR and EPDM was observed.

The solubility (S) is determined from the linear slope obtained in Figure 8a and Figure 9a as follows:

(11) $$S\left[\frac{\mathrm{mol}}{m^{3}\cdot \mathrm{MPa}}\right]=\frac{C_{\infty }\;\mathrm{slope}\;\left[\frac{\mathrm{wt}\cdot \mathrm{ppm}}{\mathrm{MPa}}\right]10^{6}\times d\left[\frac{g}{m^{3}}\right]}{m_{g}\left[\frac{g}{\mathrm{mol}}\right]}$$

where *m_g_* is the molar mass of gas used, and *d* is the density of the rubber. The permeabilities of the four gases in the NBR and EPDM polymers are obtained from the solubility and the average diffusivity by using the relation of P = D_ave_S. The permeation parameters for four gases in NBR and EPDM are summarized with those obtained by different methods in Table 1.

The values in parentheses were determined by the differential pressure method and thermal desorption analysis–gas chromatography [23] in the same specimen. The results obtained by different methods for H_2_ gas are consistent with those in the present experimental investigation within expanded uncertainty.

**Figure 10:**
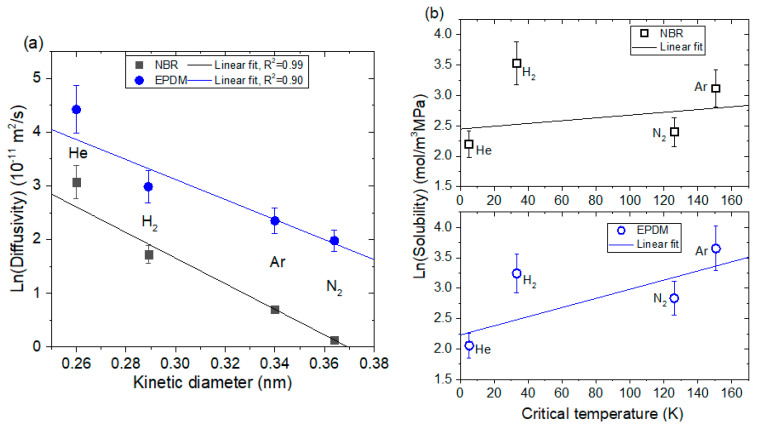
(**a**) A linear correlation between the logarithmic diffusivity and kinetic diameter and (**b**) a linear correlation between the logarithmic solubility and kinetic diameter of molecules in gas.

Differences in the permeation parameters were found for gases in both NBR and EPDM. The magnitudes of the diffusivity and permeability decrease in the orders *D*_He_ > *D*_H2_ > *D*_Ar_ > *D*_N2_ and *P*_He_ > *P*_H2_ > *P*_Ar_ > *P*_N2_ in both NBR and EPDM. Although there are many factors affecting the permeation parameters of rubber, we focus on the molecule size in the gas. The size of the permeant molecule affects the diffusivity. As the effective size of the molecule increases, the diffusivity decreases. As expected for both NBR and EPDM (Figure 10a), we found a linear correlation with a squared correlation coefficient of R^2^ > 0.90 between the logarithmic diffusivity and kinetic diameter of the molecules in the gas, which is the size of the sphere of influence that can lead to a scattering event and is also related to the mean free path of molecules in a gas [24,25].

Figure 10a also displays different diffusivity values obtained at same kinetic diameter between NBR and EPDM polymer. For the case of NBR, the existence of a –CN polar group can make it possible to increase interchain interaction, leading to the tight packing of polymer chains. As a result, the available free volume decreases, and then NBR achieves a low diffusivity of gas molecules. In contrast, EPDM could have a large free volume due to the presence of norbornene, and thus it is not easy to have a tight packing of chains, resulting in the high diffusivity of gas. In addition, EPDM chains are expected to be more flexible than NBR since the chain mobility has also been known to be governed by the chain packing characteristics.

Meanwhile, the solubility of gases depends on the relative affinity between the gas and polymer, but more strongly on the penetrant condensability correlated with the gas critical temperature (*T*_c_). The relationship between gas solubility and the critical temperature is generally expressed as [26,27]:

ln *S* = a + b*T*_c_ (12)

The constant “a” is a measure of the overall sorption capacity, while slope “b” indicates the increase in solubility with regard to the penetrant condensability. Figure 10b demonstrates the solubility of the four gases versus critical temperature for two polymers. It is observed for EPDM rather than NBR that the logarithmic solubility increases nearly linearly with the increase in the critical temperature, except for H_2_ gas, which deviates from linearity. A similar relationship was reported for polyvinylpyridine film [27].

We present the performance parameters of capacitor sensors, such as sensitivity, resolution, stability, detection range, and response time, for the two sensors in Table 2 with a related description.

The sensitivity is defined as the slope obtained by the change in capacitance with regard to the water level in the unit of ml. The sensitivity is the most important factor deciding the performance of a sensor. The coaxial-cylindrical capacitor sensor with a high sensitivity and minute resolution could be a better choice.

## 6. Conclusions

We first developed an automatic technique for determining the permeation of various gases, including H_2_, He, N_2_, and Ar. This simple and effective method combines a volumetric measurement using a graduated cylinder with water level detection by capacitance measurement with two different types of electrodes in real time. This technique is able to simultaneously evaluate three sets of diffusion characteristics of gas by quantitatively analyzing the amount of gas released after high-pressure gas charging and subsequent decompression. With the autoreading and autocontrol of temperature and pressure sensors, fluctuations due to variations in the temperature and pressure of the laboratory environment were removed, resulting in good-quality permeation data. The results achieved for polymers demonstrate that the H_2_ permeation properties determined by the developed method are in agreement with those determined by the differential pressure method and gas chromatography.

The experimental investigation indicates that the gas content emitted from the NBR and EPDM satisfied Henry’s law up to a pressure of 9 MPa, which confirmed that the content was primarily proportional to the pressure. The solubility and diffusivity were identical for all specimens employed, regardless of the sample shape and dimensions. This is a general trend, but different diffusivity values were found for thicker specimens. The different diffusivities for each gas can be attributed to the different kinetic diameters of the molecules in the gas.

In conclusion, a technique for determining permeation with capacitance measurement using a frequency response analyzer could be effectively applied for automatically evaluating the transport properties of gases in polymers and other materials for cases requiring real-time and time-consuming measurements with a slow diffusion rate. This simple technique could be applied in permeation evaluation and leakage tests for all types of gas without sample size and shape limitations.

## Figures and Tables

**Table 1:** Summary of the permeability properties for the parameters of four gases in NBR and EPDM.

Specimen	Solubility (mol/m^3^·MPa)				Diffusivity (×10^−11^ m^2^/s)				Permeability (mol/m·s·MPa, ×10^−10^)			
	H_2_	He	N_2_	Ar	H_2_	He	N_2_	Ar	H_2_	He	N_2_	Ar
NBR	34.2 (35.3)	8.96	11.0	22.5	5.60 (6.50)	21.5	1.14	2.01	19.2 (22.8)	19.3	1.25	4.53
EPDM	25.6 (26.2) [23]	7.79	17.0	38.6	19.7 (24.1) [23]	83.1	7.24	10.5	50.3 (63.1) [23]	64.8	12.3	40.4

**Table 2:** Comparison of the performance parameters of capacitor sensors.

Parameter	Coaxial-Cylindrical	Semi-Cylindrical
Sensitivity	~3 pF/mL	~1 pF/mL
Resolution	~0.5 wt·ppm	~2 wt·ppm
Stability	<10 wt·ppm	<15 wt·ppm
Detection range	~max 1000 wt·ppm for H_2_	~max 1000 wt·ppm for H_2_
Response time	<1 s	<1 s

## Data Availability

The data used to support the findings of this study are available from the corresponding author upon request.
